# Genomic Architecture and Cascade Screening Gaps in Hypertrophic Cardiomyopathy: A Real-World Analysis

**DOI:** 10.3390/jcm15114186

**Published:** 2026-05-28

**Authors:** Kaho Kato, Aki Ishikawa, Tasuku Mariya, Hidemichi Kouzu, Toshiyuki Yano, Wataru Kawaharata, Yuko Takasu, Ayana Miura, Aiko Seto, Kentaro Suda, Akihiro Sakurai

**Affiliations:** 1 Division of Genomic and Preventive Medicine, Department of Clinical Genomics, Sapporo Medical University School of Medicine, Sapporo 060-8556, Japan; katokaho@sapmed.ac.jp (K.K.);; 2 Department of Obstetrics and Gynecology, Sapporo Medical University School of Medicine, Sapporo 060-0812, Japan; 3 Division of Cardiovascular-Kidney-Metabolic Medicine, Department of Internal Medicine, Sapporo Medical University School of Medicine, Sapporo 060-0812, Japan

**Keywords:** hypertrophic cardiomyopathy, genetic testing, cascade screening

## Abstract

**Background/Objectives**: Hypertrophic cardiomyopathy (HCM) is genetically heterogeneous, involving more than 11 genes. Since HCM genetic testing was covered by Japan’s national health insurance in 2022, variant detection and the need for family-based intervention have increased, although funding is limited to symptomatic patients only. In this study, we evaluated institutional genetic testing outcomes, factors associated with pathogenic variants, and follow-up of at-risk relatives. **Methods**: We retrospectively analyzed individuals with confirmed or suspected HCM who underwent genetic testing between October 2022 and June 2025. Data regarding molecular results, family history of cardiomyopathy or sudden cardiac death in first-, second-, and third-degree relatives, and cascade screening were collected. Statistical analysis was performed using R version 2025.09.2 + 418. **Results**: Among 33 probands (median age, 54 years; 51% male), 13 individuals (39%) had pathogenic or likely pathogenic variants (PV or LPV), while six (18%) harbored variants of uncertain significance (VUS), and 14 (43%) yielded negative results. The PV or LPV cohort was significantly younger at the time of testing (median, 34 vs. 59 years; *p* = 0.008) and had a family history of PV or LPV (77% vs. 20%; *p* = 0.005). Only three relatives from two PV or LPV probands underwent cascade genetic screening; two tested positive and initiated targeted cardiac surveillance. **Conclusions**: Despite achieving actionable results, the restricted uptake of cascade screening highlights the need for improved communication and systemic support to facilitate family-based testing and precision medicine.

## 1. Introduction

Hypertrophic cardiomyopathy (HCM) is a predominantly heritable cardiac disorder with a global prevalence of approximately 1 in 500 adults [1]. It is defined by unexplained left ventricular (LV) hypertrophy, specifically a wall thickness ≥15 mm as identified via echocardiography or magnetic resonance imaging [2]. Clinical manifestations are heterogeneous, ranging from asymptomatic presentations to lethal arrhythmias, chest pain, heart failure, and sudden cardiac death. Early identification of asymptomatic individuals enables longitudinal surveillance and risk stratification, facilitating lifestyle management and timely interventions such as implantable cardioverter-defibrillator (ICD) implantation, which has been shown to reduce the risk of sudden cardiac death in HCM [3].

As a genetically heterogeneous condition, HCM involves mutations in over 11 identified genes [4], primarily following an autosomal dominant inheritance pattern. Variants in myocardial myosin binding protein C (*MYBPC3*) and cardiac myosin heavy chain (*MYH7*) genes, which encode critical sarcomere proteins, constitute approximately 70–80% of identified cases [5,6]. In cohorts of HCM patients, pathogenic variants are currently detected in roughly 34% of index cases [7], highlighting the significant proportion of cases that remain genetically unexplained.

Genetic testing facilitates diagnostic clarification, prognostic estimation, and familial risk stratification through cascade genetic testing and cardiac screening. The 2024 Joint Guidelines from the Japanese Circulation Society, the Japanese College of Cardiology, and the Japanese Society of Pediatric Cardiology and Pediatric Surgery (JCS/JCC/JSPCCS) assign Class I recommendations to genetic counseling and testing for HCM patients [8]. Furthermore, these guidelines recommend genetic screening for relatives of probands harboring pathogenic variants and clinical evaluation for all first-degree relatives [8].

Since the 2022 inclusion of genetic testing for HCM under the national health insurance of Japan, access to genetic testing may have improved, potentially increasing the identification of pathogenic variants and the need for familial intervention, but testing of asymptomatic at-risk relatives remains unfunded. However, empirical data regarding the implementation of this testing in Japanese clinical practice remain scarce. In this study, we evaluate the prevalence of pathogenic variants and associated factors for variant detection and examine institutional protocols for the follow-up of at-risk relatives.

## 2. Materials and Methods

Probands were defined as individuals undergoing genetic evaluation for cardiomyopathy. After reimbursement began, they were referred for this evaluation during inpatient cardiomyopathy evaluation or in the outpatient clinic when testing was considered clinically appropriate by the treating cardiologists with expertise in cardiomyopathy. Between October 2022 and June 2025, 38 probands were screened at Sapporo Medical University Hospital. Five individuals were excluded: four due to a dilated cardiomyopathy diagnosis and one for not meeting HCM diagnostic criteria. The final cohort comprised 33 probands with confirmed or suspected HCM. Diagnoses were established via medical history, clinical examination, 12-lead electrocardiography (ECG), and echocardiography. Secondary cardiomyopathies were systematically excluded through these modalities.

HCM diagnosis was based on maximal LV wall thickness, assessed by echocardiography or cardiac magnetic resonance imaging. HCM was defined as a maximal LV wall thickness ≥15 mm, or ≥13 mm in individuals with a family history of HCM. Phenotypic classification was performed as follows: hypertrophic obstructive cardiomyopathy was defined by a left ventricular outflow tract (LVOT) gradient ≥30 mmHg at rest, or <30 mmHg at rest but ≥30 mmHg with provocation; nonobstructive HCM was defined as an LVOT gradient <30 mmHg at rest or with provocation; apical HCM was defined as hypertrophy predominantly localized to the LV apex; and dilated-phase HCM was defined as LV systolic dysfunction (ejection fraction <50%) in patients with a prior diagnosis of HCM. Cases not meeting diagnostic criteria at the initial evaluation but fulfilling criteria during longitudinal follow-up based on echocardiography or cardiac magnetic resonance imaging were included. We also determined the indications for an ICD based on the guidelines of the Japanese Circulation Society and the Japanese Heart Failure Society [2].

We retrospectively reviewed electronic health records to aggregate data regarding the demographic and clinical characteristics of the probands, molecular results, and familial history of HCM or sudden cardiac death in first-, second-, and third-degree relatives. Additionally, the implementation of cascade screening among relatives was evaluated.

During pre-test genetic counseling, probands or parents of pediatric probands were informed about the inheritance patterns of HCM and the clinical relevance of cascade screening. Following disclosure of genetic test results, post-test counseling included recommendations for cascade testing or clinical evaluation for at-risk relatives, depending on the proband’s results. They were provided with contact information to facilitate referral of family members to our clinic. No standardized written materials or family communication letters were provided to support the dissemination of genetic information to relatives. In pediatric probands, parental genetic testing was offered as part of cascade screening following disclosure of genetic test results.

Molecular evaluation was conducted using a 25-gene next-generation sequencing panel at a certified laboratory. The panel targeted primary HCM-associated genes, including *ACTC1*, *ACTN2*, *CSRP3*, *JPH2*, *MYBPC3*, *MYH7*, *MYL2*, *MYL3*, *PLN*, *TNNC1*, *TNNI3*, *TNNT2*, *TPM1*, *ALPK3*, *CACNA1C*, *DES*, *FHL1*, *FLNC*, *GLA*, *LAMP2*, *PRKAG2*, *PTPN11*, *RAF1*, *RIT1*, and *TTR.* Variants were classified using information from publicly available databases, including ClinVar and the Human Gene Mutation Database (HGMD). Variants annotated as “disease-causing mutation” in the HGMD were considered to correspond to pathogenic or likely pathogenic classifications, while ClinVar classifications were used as reported. For the purposes of this study, the term “pathogenic variants” refers to pathogenic and likely pathogenic variants unless otherwise specified [9]. The integrity of variant confirmation and clinical interpretation was rigorously validated by a panel of clinical geneticists.

Data were analyzed using R version 4.3.1 (R Foundation for Statistical Computing, Vienna, Austria), and continuous variables are reported as the median (interquartile range [IQR]). The Wilcoxon rank sum test was employed for median comparisons, while categorical variables were assessed via Fisher’s exact test, and statistical significance was defined by a two-tailed *p* < 0.05. Due to the limited sample size, multivariable analysis was not performed, so all analyses were exploratory and based on univariable comparisons without adjustment for potential confounders.

## 3. Results

**Figure 1:**
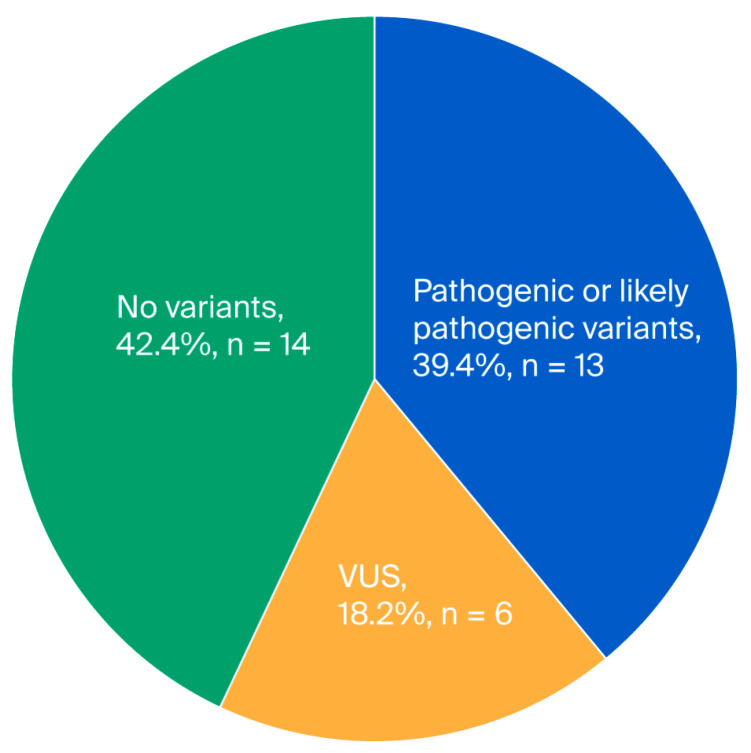
Detection rate in genetic testing (*n* = 33).

The demographic and clinical characteristics of the cohort are summarized in Table 1. The cohort comprised 17 males (51.2%), and the probands exhibited a median age of 40 [IQR, 27–57] years at diagnosis. Phenotypically, nonobstructive HCM was present in 18 patients (54.5%), while 12 (36.3%) presented with obstructive HCM. Implantable cardioverter-defibrillators (ICDs) were utilized in eight cases (24.2%), of which seven were for primary prevention, and one was for secondary prevention. Molecular analysis identified pathogenic or likely pathogenic variants in 13 probands (39.4%), VUS in six (18.2%), and no variants in 14 (42.4%) (Figure 1). Among the 13 pathogenic cases, *MYBPC3* predominated (*n* = 9), with *MYH7*, *MYL3*, *TNNI3*, and *TNNT2* each identified in single instances (Table 2 and Table S1).

We compared clinical characteristics between cases with the pathogenic variants and those with VUS or negative variants. There were no statistically significant differences in the distribution of obstructive versus nonobstructive HCM, syncope, nonsustained ventricular tachycardia, or maximum LV wall thickness (Table 3). A more detailed comparison across phenotype subtypes and echocardiographic parameters likewise showed no significant difference (Table S2). Similarly, the prevalence of ICD implantation was not significantly different between groups (5/13 vs. 3/20; *p* = 0.213; Table 3), although a higher proportion was observed in the pathogenic group.

The median age at genetic evaluation was significantly lower in the pathogenic group than in the VUS/negative group (34 [IQR, 20–51] vs. 59 [IQR, 48–64] years; *p* = 0.008; Table 3), corresponding to an approximate median difference of 25 years. A familial history of HCM or sudden cardiac death was reported in 14 probands (42.4%; Table 1). Remarkably, the pathogenic variant cohort exhibited a significantly higher prevalence of positive family history (10 of 13; 76.9%) than the combined cohort of individuals with VUS or negative results (4 of 20; 20.0%; *p* = 0.003), corresponding to an odds ratio of 12.0 (95% CI, 2.0–103.7).

Cascade genetic screening was performed for three relatives from two probands harboring pathogenic variants (Table 3). The median age of these screened individuals was 7 years (range, 6–9), and all were first-degree relatives, specifically two siblings and one child of the respective probands. Genetic evaluation identified the familial variant in two relatives, who subsequently underwent assessment by pediatric cardiologists. Initial clinical examinations revealed no phenotypes consistent with HCM; however, both individuals were enrolled in a protocol for annual cardiac surveillance. No parents of pediatric probands underwent genetic testing at our institution despite being offered cascade screening. In addition, no relatives of probands lacking pathogenic variants sought clinical or cardiac screening at our hospital.

## 4. Discussion

This investigation reveals that 39.4% of HCM patients harbored pathogenic or likely pathogenic variants, primarily within *MYBPC3*. Significant associations were identified between variant positivity and both younger age at evaluation and positive family history. Conversely, cascade screening uptake among at-risk relatives remained low at 15.4%. This study provides a critical real-world analysis of clinical practice following the 2022 inclusion of HCM genetic testing under the national health insurance system of Japan, although coverage remains limited to individuals with symptomatic or suspected HCM, and not cascade testing of asymptomatic relatives at-risk.

The diagnostic yield and genetic architecture observed in this cohort align with established data from Japanese and Western populations, where pathogenic sarcomeric variants are identified in 37–47% of cases [6,10,11,12]. The marked predominance of *MYBPC3* alongside a lower frequency of *MYH7* corresponds with documented regional variations within Japan. Specifically, northeastern regions exhibit a higher prevalence of *MYBPC3* alterations [13]. These findings validate that our real-world cohort accurately reflects the established genomic patterns of HCM.

Elucidating geographical variation is critical for informing tailored genetic counseling and clinical management. Aggregating clinical data from regions with distinct variant patterns may clarify gene-specific risks, enhance the interpretation of geographically enriched alleles, and facilitate the development of region-specific frameworks for precision cardiovascular care.

Lower age at evaluation and a positive family history correlate with increased variant detection, aligning with the established literature [5,14]. However, the utility of self-reported family history is constrained by incomplete familial medical knowledge and variable penetrance of sarcomeric genes [7,15]. Consequently, the broad implementation of genetic testing in individuals with HCM remains essential, as recommended by current guidelines, to identify asymptomatic carriers and optimize clinical management.

By contrast, genotype-positive cases did not demonstrate a significant difference in phenotypic severity compared with genotype-negative or VUS cases across multiple clinical parameters. Although the ICD was more frequently implanted in the pathogenic group, this did not reach statistical significance. These findings may reflect limited statistical power; they are also consistent with prior observations that genotype–phenotype correlations in HCM are heterogeneous with variable expressivity and incomplete penetrance, thereby limiting the ability to predict clinical severity and outcomes at individual levels [16].

Cascade genetic testing facilitates the early identification of at-risk, asymptomatic relatives, providing a significant clinical advantage. Timely diagnosis followed by the initiation of appropriate management protocols improves cardiac outcomes [17,18]. In addition to clinical utility, cascade screening has been reported to be cost-effective by enabling targeted surveillance of variant carriers while reducing unnecessary long-term clinical follow-up in non-carriers [19]. Consequently, the 2024 JCS/JCC/JSPCCS guidelines strongly recommend genetic screening for all first-degree relatives of probands harboring pathogenic variants [8].

Despite established clinical guidelines, cascade screening uptake within our cohort appeared low. The uptake reported here reflects evaluations performed at our institution and should therefore be interpreted as a minimum estimate, as some relatives may have undergone assessment at other centers. Only one in six probands harboring pathogenic variants initiated genetic screening in their families, resulting in just three relatives being evaluated. Notably, all individuals who underwent cascade testing were minors, and no adult relatives presented for evaluation. Given that genetic testing in children is typically parent-directed, this finding may reflect different barriers to uptake between adult and pediatric relatives. This pattern contrasts with prior studies reporting higher uptake across a broader range of relatives. For instance, a prior report from the United States indicates that 52.5% of at-risk relatives received risk disclosure, with nearly half of those undergoing cascade testing, with higher uptake observed among parents and children [20]. Another study, from the Netherlands, reports that 60% of relatives attended genetic counseling within 6 months of disclosure to probands, particularly among symptomatic individuals or those with a family history of sudden death at a young age [21].

Although we did not directly assess the reasons for the diminished uptake observed in our cohort, several factors reported in prior studies may be relevant. One potential factor is insufficient communication of genetic findings from probands to relatives. Sharing results is frequently influenced by factors such as communication frequency, geographical distance, relationship quality, and emotional proximity [20]. In this context, genetic counseling may benefit from assessing probands’ intentions to communicate results and providing support that accounts for family dynamics. Another possible factor is reluctance among relatives to undergo counseling or molecular testing. Previously reported barriers included the limited treatment options for HCM and the significant financial burden associated with longitudinal medical care and genetic testing [22]. A lack of awareness regarding HCM symptoms, prognosis, and inheritance patterns may also reduce motivation for familial testing.

The financial burden remains a primary impediment to cascade screening in Japan, as genetic evaluation for asymptomatic relatives is excluded from national health insurance coverage. Furthermore, longitudinal surveillance of variant carriers, including periodic clinical assessments, must be funded at personal expense. These economic constraints likely deter at-risk individuals from pursuing molecular testing and participating in essential preventive care protocols.

Concerns related to genetic discrimination remain a potential driver of the low uptake of cascade screening. A Japanese national survey identified public concern regarding disadvantages in insurance enrollment, employment, and matrimonial prospects related to the use of genomic data [23]. In response, both the Life Insurance Association of Japan and the General Insurance Association of Japan issued 2022 declarations affirming that genetic test results and familial medical history are not utilized in insurance underwriting, and the Genomic Medicine Promotion Act was enacted in 2023 [24,25,26]. The sustained development and rigorous implementation of these regulatory frameworks are essential to mitigate discriminatory fears and facilitate broader acceptance of cascade genetic screening among at-risk families, ensuring that legal protections translate into clinical participation. However, these explanations remain speculative, as they were not directly evaluated in this cohort.

Despite the structural and societal barriers, targeted clinical interventions remain essential to facilitate cascade genetic screening. Educational resources, such as disease-specific brochures, web-based information, and family letters recommending counseling, may streamline communication and increase participation. Furthermore, longitudinal follow-up with probands and periodic updates of family history can provide necessary stimuli for relative testing. Future investigations should employ survey- and interview-based approaches to investigate factors contributing to the low uptake of cascade screening, including family communication patterns, motivation and willingness of at-risk relatives to undergo testing, and systemic barriers to genetic testing access.

The National Society of Genetic Counselors recommends clinical cardiac screening among first-degree relatives of probands harboring VUS or those without identifiable variants, reflecting the incomplete characterization of the genetic architecture of HCM [27]. Although no relatives in these categories underwent cardiac evaluation at our institution, the overall cascade screening uptake reported in this study may be underestimated, as some relatives may have undergone evaluation at other medical facilities. In addition, routine health examinations in Japan, which may include electrocardiography, provide alternative opportunities for cardiac assessment among at-risk individuals. Systematic and regular updates on relatives’ cardiac evaluations would enable genetic professionals to identify high-risk individuals requiring further assessment and refine longitudinal clinical management strategies.

This study has distinct strengths and limitations. First, this study was performed at a hospital using real-world data, reflecting contemporary practice following the reimbursement of genetic testing in Japan. However, the small sample size precluded multivariable adjustment; therefore, the findings should be considered exploratory and may be subject to confounding factors. Such a sample size from one medical facility limits the generalizability of our results to broader HCM populations. Additionally, family history was directly collected from patients, which might have introduced bias, potentially omitting affected relatives with HCM or including inaccurate data about relatives’ health status.

Since the inclusion of HCM genetic testing under the national health insurance system of Japan in 2022, access to genetic testing for symptomatic patients may have improved, potentially facilitating the identification of individuals with pathogenic variants, although this effect could not be directly evaluated in the present study, and the funding does not cover asymptomatic at-risk relatives. In addition, the integration of whole-exome and whole-genome sequencing into routine clinical practice is anticipated to further expand detection via secondary findings. Furthermore, the introduction of CAMZYOS^®^ (mavacamten) in Japan in 2025 represents a pivotal shift toward precision therapeutics for HCM [28]. Given the lifelong nature of HCM, continued follow-up and proactive management are essential. Strengthening collaboration among cardiologists, genetic professionals, and multidisciplinary teams is essential to enhance diagnostic accuracy through genetic testing, facilitate effective cascade screening for at-risk relatives, and ultimately optimize long-term clinical outcomes for managing both affected individuals and their families.

## 5. Conclusions

In the present retrospective, single-center analysis, we identified pathogenic variants in 39.4% of HCM cases, characterized by a predominance of *MYBPC3* and a relatively low frequency of *MYH7*. Diagnostic yield was significantly associated with younger age at the time of genetic evaluation and a documented family history. Conversely, the uptake of cascade genetic screening among at-risk relatives remained remarkably low, with only 15.4% of variant-positive probands facilitating familial testing. Notably, no at-risk adult relatives underwent cascade genetic testing, and all individuals who were tested were children, with testing initiated by their parents. Further research is warranted to explore the communication dynamics between probands and their at-risk relatives as well as the motivations and barriers influencing screening participation. Our research underscores significant opportunities to refine HCM management by expanding data on genetic variants and implementing strategies to enhance family screening.

## Figures and Tables

**Table 1:** Clinical characteristics of all participants.

Clinical Characteristics	All (*n* = 33)
Age at diagnosis	40 (27–57)
Age at genetic testing	54 (34–61)
Male, *n* (%)	17 (51.2)
Family history of HCM and/or sudden death, *n* (%)	14 (42.4)
Phenotype	
Hypertrophic nonobstructive cardiomyopathy, *n* (%)	18 (54.5)
Hypertrophic obstructive cardiomyopathy, *n* (%)	12 (36.3)
Apical HCM, *n* (%)	2 (6.1)
Dilated phase of HCM, *n* (%)	1 (3.0)
Syncope, *n* (%)	6 (18.2)
Nonsustained ventricular tachycardia, *n* (%)	14 (42.4)
Echocardiographic	
Left atrial diameter, mm	38.7 (34.2–41.2)
Interventricular septum thickness, mm	13.3 (11.1–15.6)
Posterior LV * wall thickness, mm	10.3 (8.9–11.2)
LV * diameter at end diastole, mm	42.3 (39.9–45.2)
LV * diameter at end systole, mm	25.6 (23.3–28.3)
Ejection fraction, %	62.4 (59.9–66.5)
Maximum LV * wall thickness, mm	16.1 (14.0–18.8)
Implantable cardioverter defibrillator, *n* (%)	8 (24.2)

* LV, left ventricle.

**Table 2:** List of pathogenic variants.

No.	Gene	Reference	Nucleotide Change	Amino Acid Change	Number of Probands
1	*MYBPC3*	NM_000256.3	2285T > A	Val762Asp	1
2	*MYBPC3*	NM_000256.3	1621C > T	Gln541 *	1
3	*MYBPC3*	NM_000256.3	1777del	Ser593Profs * 9	2
4	*MYBPC3*	NM_000256.3	237C > G	Tyr79 *	1
5	*MYBPC3*	NM_000256.3	1505G > A	Arg502Gln	1
6	*MYBPC3*	NM_000256.3	2459G > A	Arg820Gln	1
7	*MYBPC3*	NM_000256.3	2479C > T	Gln827 *	1
8	*MYBPC3*	NM_000256.3	3190 + 5G > A		1
9	*MYH7*	NM_000257.3	746G > A	Arg249Gln	1
10	*MYL3*	NM_000258.2	466T > C	Met149Thr	1
11	*TNNT2*	NM_001001430.2	487G > A	Glu163Lys	1
12	*TNNI3*	NM_000363.5	592C > G	Leu198Val	1

* indicates a stop codon.

**Table 3:** Comparison of clinical characteristics between cases with pathogenic or likely pathogenic variants and cases without them.

Demographics	Pathogenic or Likely Pathogenic Variants (*n* = 13)	No Variants or VUS (*n* = 20)	*p*-Value
Age at diagnosis (years)	27 (16–34)	50 (34–61)	0.003
Age at genetic testing (years)	34 (20–51)	59 (48–64)	0.008
Male, *n* (%)	5 (38.5)	12 (60.0)	0.296
Family history of HCM and/or sudden death, *n* (%)	10 (76.9)	4 (20.0)	0.003
Phenotype			0.442
Hypertrophic nonobstructive cardiomyopathy, *n* (%)	8	10	
Hypertrophic obstructive cardiomyopathy, *n* (%)	3	9	
Syncope, *n* (%)	1	5	0.364
Nonsustained ventricular tachycardia, *n* (%)	6	8	1
Maximum left ventricle wall thickness, mm	16.2 (15.8–18.4)	15.1 (14.0–19.1)	0.278
Implantable cardioverter defibrillator, *n* (%)	5	3	0.213
Cascade genetic screening, *n* (%)	2 (15.4)	-	-

## Data Availability

Deidentified individual participant data will be shared upon reasonable request to the corresponding authors.

## Supplementary Materials

The following supporting information can be downloaded at: https://www.mdpi.com/article/10.3390/jcm15114186/s1. Table S1. List of pathogenic variants and evidence summary. Table S2. Comparison of phenotype and echocardiographic parameters between cases with pathogenic or likely pathogenic variants and cases without them.

## Abbreviations

The following abbreviations are used in this manuscript:

HCM	Hypertrophic Cardiomyopathy
VUS	Variant of Uncertain Significance
LV	Left Ventricular
ECG	12-Lead Electrocardiography
