# Supplementary material for: Genomic Architecture and Cascade Screening Gaps in Hypertrophic Cardiomyopathy: A Real-World Analysis
Source: J Clin Med. 2026 May 28;15(11):4186. doi: 10.3390/jcm15114186 (PMC13258485; doi:10.3390/jcm15114186)
Supplement: Supplementary file 1 [file jcm-15-04186-s001.zip › jcm-4228682-supplementary.pdf]

Table S1 List of pathogenic variants and evidence summary

No	Gene	Reference	Nucleotide change	Amino acid change	Variant type	HGMD	ClinVar	Evidence summary	Number of probands
1	*MYBPC3*	NM_000256.3	2285T>A	Val762Asp	Missense	Disease causing	Pathogenic/Likely Pathogenic	Reported in databases	1
2	*MYBPC3*	NM_000256.3	1621C>T	Gln541*	Nonsense	Disease causing	Pathogenic	Predicted loss-of-function	1
3	*MYBPC3*	NM_000256.3	1777del	Ser593Profs*9	Frame shift	Disease causing	Pathogenic	Predicted loss-of-function	2
4	*MYBPC3*	NM_000256.3	237C>G	Tyr79*	Nonsense	Disease causing	Pathogenic	Predicted loss-of-function	1
5	*MYBPC3*	NM_000256.3	1505G>A	Arg502Gln	Missense	Disease causing	Pathogenic/Likely Pathogenic	Reported in databases	1
6	*MYBPC3*	NM_000256.3	2459G>A	Arg820Gln	Missense	-	Pathogenic/Likely Pathogenic	Reported in databases	1
7	*MYBPC3*	NM_000256.3	2479C>T	Gln827*	Nonsense	Disease causing	-	Predicted loss-of-function	1
8	*MYBPC3*	NM_000256.3	3190+5G>A		Intron	Disease causing	Pathogenic/Likely Pathogenic	Predicted splice-site alteration	1

9	*MYH7*	NM_000257.3	746G>A	Arg249Gln	Missense	Disease causing	Pathogenic/Likely Pathogenic	Reported in databases	1
10	*MYL3*	NM_000258.2	466T>C	Met149Thr	Missense	Disease causing	Conflicting classifications of pathogenicity Likely pathogenic(1); Uncertain significance(3)	Reported in databases	1
11	*TNNT2*	NM_001001430.2	487G>A	Glu163Lys	Missense	Disease causing	Conflicting classifications of pathogenicity Pathogenic(2); Likely pathogenic(2); Uncertain significance(1)	Reported in databases	1
12	*TNNI3*	NM_000363.5	592C>G	Leu198Val	Missense	Disease causing	Pathogenic/Likely Pathogenic	Reported in databases	1

Table S2 Comparison of phenotype and echocardiographic parameters between cases with pathogenic or likely pathogenic variants and cases without them

Demographics	Pathogenic or likely pathogenic variants (n=13)	No variants or VUS (n=20)	p-value
Phenotype			0.397
Hypertrophic nonobstructive cardiomyopathy, n	8	10	
Hypertrophic obstructive cardiomyopathy, n	3	9	
Apical HCM, n	1	1	
Dilated phase of HCM, n	1	0	
Echocardiographic			
Left atrial diameter, mm	38.9 (33.2-41.9)	37.8 (34.8-41.0)	0.922
Interventricular septum thickness, mm	13.4 (12.4-15.1)	13.1 (10.5-16.3)	0.599
Posterior LV wall thickness, mm	9.4 (8.1-10.6)	10.5 (9.0-11.3)	0.106
LV diameter at end diastole, mm	43.5 (39.8-45.8)	42.0 (39.9-44.5)	0.533
LV diameter at end systole, mm	24.5 (21.9-26.8)	26.5 (23.5-28.7)	0.115
Ejection fraction, %	60.8 (55.5-67.8)	62.7 (60.0-65.6)	0.893
